# Xanthohumol Alters Gut Microbiota Metabolism and Bile Acid Dynamics in Gastrointestinal Simulation Models of Eubiotic and Dysbiotic States

**DOI:** 10.3390/ijms262110698

**Published:** 2025-11-03

**Authors:** Paige E. Jamieson, Nicholas J. Reichart, Claudia S. Maier, Thomas J. Sharpton, Ryan Bradley, Thomas O. Metz, Jan F. Stevens

**Affiliations:** 1 College of Health, Oregon State University, Corvallis, OR 97331, USA; jamiesop@oregonstate.edu; 2 Linus Pauling Institute, Oregon State University, Corvallis, OR 97331, USA; claudia.maier@oregonstate.edu; 3 Earth and Biological Sciences Directorate, Pacific Northwest National Laboratory, Richland, WA 99352, USA; nicholas.reichart@pnnl.gov (N.J.R.); thomas.metz@pnnl.gov (T.O.M.); 4 Department of Chemistry, Oregon State University, Corvallis, OR 97331, USA; 5 Department of Microbiology, Oregon State University, Corvallis, OR 97331, USA; thomas.sharpton@oregonstate.edu; 6 Department of Statistics, Oregon State University, Corvallis, OR 97331, USA; 7 Helfgott Research Institute, National University of Natural Medicine, Portland, OR 97201, USA; rybradley@health.ucsd.edu; 8 Herbert Wertheim School of Public Health, University of California, San Diego, CA 92093, USA; 9 Department of Pharmaceutical Sciences, Oregon State University, Corvallis, OR 97331, USA

**Keywords:** polyphenol, phytochemical, gut microbiota, dysbiosis, bile acids, SHIME, xanthohumol, *Humulus lupulus*

## Abstract

Xanthohumol (XN), a polyphenol from hops (*Humulus lupulus*), exhibits antioxidant, anti-inflammatory, antihyperlipidemic, and chemo-preventive activity. Preclinical evidence suggests gut microbiota are critical to mediating some of these bioactivities. Nevertheless, its precise impact on human gut microbiota, particularly at supplemental doses, remains poorly characterized. We evaluated 200 mg/day XN for 3 weeks on human gut microbiota in a eubiotic and dysbiotic model using the Simulator of the Human Intestinal Microbial Ecosystem (SHIME^®^). Functional assessments of microbiota included quantification of XN metabolites, short-chain fatty acids (SCFAs), and untargeted metabolomics of the digestive metabolome. Bacterial composition was assessed by 16S rRNA gene sequencing. XN reduced alpha-diversity and short-chain fatty acid production in both models, as well as altered taxa abundance variably between models. XN disrupted bile acid metabolism through inhibition of microbial bile salt hydrolase (BSH). The modulation of bile acid metabolism has important implications for host-level bioactivity of XN.

## 1. Introduction

Xanthohumol (XN) is a prenylated chalcone found in the hop flower (*Humulus lupulus*), with established anti-inflammatory, anti-hyperlipidemic, anti-hyperglycemic, and chemo-preventive properties [1,2,3,4]. As a constituent in beer, dietary intake is minimal; however, it has emerged as a functional ingredient for dietary supplements due to its diverse suite of bioactivity. XN-enriched hop extracts are currently produced commercially by the hop processing industry and are creating a market for hops outside the beer industry [5].

Several studies have reported that XN exerts anti-inflammatory effects through its interaction with the Keap1-Nrf2 pathway and the NF-κB signaling pathway [6,7,8]. Preclinical studies of metabolic and inflammation-driven disease models have shown particular promise [1,2,9]. For example, when administered to mice fed a high-fat diet (HFD), XN attenuates obesity-related metabolic impairments, including glucose and lipid dysregulation, systemic inflammation, and insulin resistance [10,11]. Interestingly, XN is relatively poorly absorbed, and low correlation between circulating levels and observed bioactivity has prompted investigations into its interactions with the gut microbiota as mediating some of its biological effects [12,13]. Notably, while conventional mice fed a HFD with XN were protected against obesity-related impairments, these protections were lost in germ-free mice, highlighting a critical role of the gut microbiome in XN’s effect [4,14]. Furthermore, XN modulates microbial composition in intestinal disease models with gut microbiome dysbiosis. Mice supplemented with XN were protected from inflammation, intestinal damage, and gut microbiome disruptions, in an inflammatory bowel disease model, resembling healthy controls [15,16].

Despite these findings, the precise role of the gut microbiome in shaping XN’s bioactivity is unknown. The gut microbiota contributes to host physiology largely through the production of bioactive microbial metabolites, and XN has been shown to influence microbial community structure and metabolic function [4]. XN inhibits the growth of certain anaerobic pathogens *in vitro*, including *Bacteroides fragilis*, *Clostridium perfringens*, and *Clostridioides difficile*, while animal models demonstrate that it may also promote beneficial taxa [4,17]. Importantly, gut microbes can metabolize XN into structurally and functionally distinct compounds [18,19]. For example, microbial reduction of the α,β-unsaturated carbonyl group produces α,β-dihydroxanthohumol (DXN), a derivative with enhanced microbiota-modulating capacity [4]. Collectively, the bidirectional interactions between gut microbes and XN influences its potency, inter-individual variability, and physiological relevance of XN.

Despite evidence supporting XN’s host-level health-promoting effects, mechanisms mediated by gut microbes remain unclear. To address this, we employed the Simulator of the Human Intestinal Microbial Ecosystem (SHIME^®^), a validated, long-term, anaerobic gut simulation model that mimics colonic conditions for microbial fermentation experiments [20,21]. This ex vivo system overcomes key challenges associated with resolving spatially distinct microbial activities in vivo, allowing for controlled and compartment-specific analyses We investigated a supplemental dose—comparable to those used in previous animal studies [4]—to evaluate its impact on human gut microbiota. Two parallel SHIME^®^ models were employed separately to investigate XN’s effect on functionally divergent microbial communities: healthy and disease-related dysbiosis.

## 2. Results

### 2.1. Gut Microbiota-Derived Metabolism of XN Varies by Colon Compartment and Time

**Figure 1:**
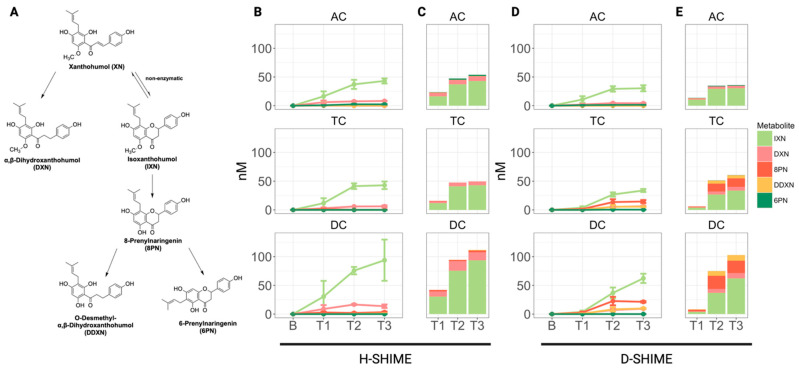
Microbiota-derived metabolites of XN by colon compartment. (**A**) Microbial metabolism of XN. Metabolite generation increased over time and along distal compartments in both model systems (**B**,**D**). Data points represent mean measurements from each model. Error bars represent the standard deviation. Stacked bar charts show metabolite composition at each time point (**C**,**E**). Measurements were performed in triplicate. AC: ascending colon; TC: transverse colon; DC: descending colon; B: baseline; T1: treatment week 1; T2: treatment week 2; T3: treatment week 3.

Each SHIME model (healthy: H-SHIME, dysbiosis: D-SHIME) consisted of five vessels, representing the stomach (ST), small intestine (SI), ascending colon (AC), transverse colon (TC), and descending colon (DC). All samples were collected from the colon reactors. Isoxanthohumol (IXN), α,β-dihydroxanthohumol (DXN), 8-prenylnaringenin (8PN), *O*-desmethyl-α,β-dihydroxanthohumol (DDXN), and 6-prenylnaringenin (6PN) were found in both models (Figure 1). A similar trend of increasing metabolite concentration was observed from the AC to DC vessels in both models, suggesting increasing metabolic capacity of the microbial community distally along the colon. This observation is supported by our previous work in humans [22] where DXN was detected in stool at higher concentrations compared to the trace amounts found in plasma and 24 h urinary collections, suggesting increased reductive metabolism in the distal colon. Additionally, we observed a similar trend of increasing metabolite concentration over the successive treatment weeks in both SHIME models, suggesting microbial communities adapt to XN exposure by increasing metabolic capacity. Collectively, our findings support previous work suggesting that human gut microbiota generally do not support the existence of strict metabotypes but have variable ability to reduce XN through the DXN pathway, as well as to transform XN through the 8PN to DDXN pathway. Additionally, a subset of samples was examined for the presence of the following potential XN metabolites as their 3NPH derivatives: 4-hydroxyphenylpropionic acid, 4-hydroxyphenylacetic acid, and 4-hydroxybenzoic acid. These analytes were detected in reaction mixtures containing standards and 3NPH, but they could not be detected in the biological samples, indicating that these putative metabolites were not formed.

### 2.2. XN Reduces Short-Chain Fatty Acid Concentration

**Figure 2:**
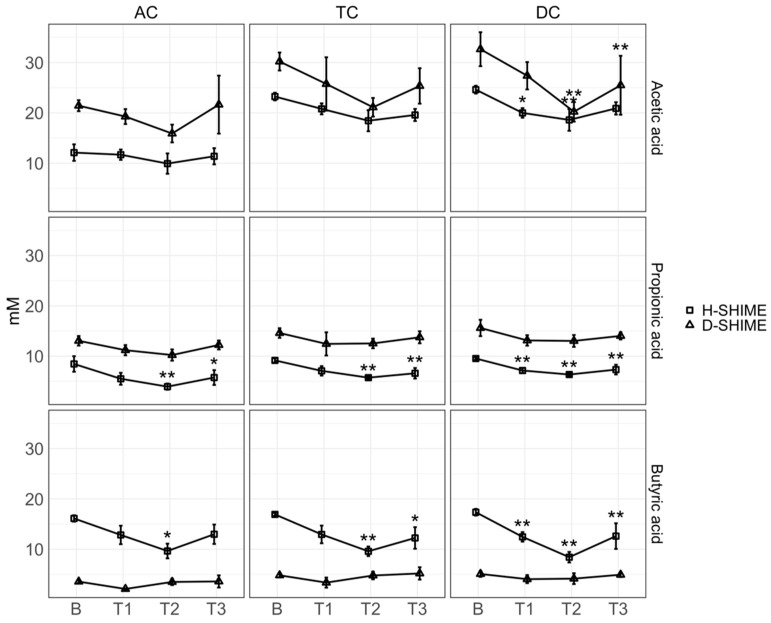
XN significantly reduces SCFA formation in colonic SHIME compartments. Greater changes in SCFA concentration were observed in H-SHIME reactors, where acetic acid, propionic acid, and butyric acid decreased compared to baseline. Within the D-SHIME, acetic acid only significantly decreased in the DC compartment compared to baseline. Significant changes within each SHIME model were determined by one-way ANOVA followed by post hoc Bonferroni analysis to compare against baseline. Shapes represent mean measurements from each model. Measurements were performed in triplicate. Error bars represent the standard deviation. AC: ascending colon; TC: transverse colon; DC: descending colon; B: baseline; T1: treatment week 1; T2: treatment week 2; T3: treatment week 3. *, *p* ≤ 0.05; **, *p* ≤ 0.01.

To explore XN supplementation on microbial saccharolytic fermentation, SCFA (acetic acid, propionic acid, and butyric acid) concentrations were assessed. Generally, XN exposure resulted in a reduction in SCFA concentration across both models (Figure 2). A reduction in acetic acid was observed in the DC compartment of both models compared to baseline (H-SHIME: *p* = 0.05, *p* = 0.005, and *p* = 0.05 for T1, T2, and T3, respectively; D-SHIME: *p* = 0.003 and *p* = 0.003, T2 and T3, respectively). Additionally, XN significantly reduced propionic acid and butyric acid among all colon compartments of the H-SHIME. We observed no significant effect of XN on production of propionic acid and butyric acid in the dysbiosis model (D-SHIME). Notably, a large difference in baseline SCFA levels was observed between models with lower butyric acid in the D-SHIME characteristic of Crohn’s disease dysbiosis. These findings suggest that XN may have a greater effect on SCFA reduction in healthy microbial communities with greater taxonomic diversity compared to dysbiotic communities.

### 2.3. XN Alters Gut Microbiota Community Structure

**Figure 3:**
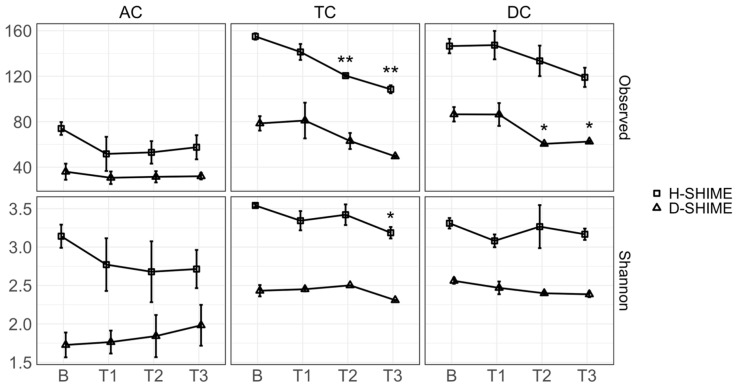
XN significantly reduces alpha diversity of colon microbiota. Observed richness (at T2 and T3) and Shannon’s entropy (at T3) was significantly reduced in the TC compartment of the H-SHIME compared to baseline. Observed richness (at T2 and T3) was significantly reduced in the DC compartment of the D-SHIME compared to baseline. Significant changes within each SHIME model were determined by one-way ANOVA followed by post hoc Bonferroni analysis compared to baseline. Shapes represent mean measurements from each model. Measurements were taken in triplicate. Error bars represent the standard deviation. AC: ascending colon; TC: transverse colon; DC: descending colon; B: baseline; T1: treatment week 1; T2: treatment week 2; T3: treatment week 3. *, *p* ≤ 0.05; **, *p* ≤ 0.01.

After filtering for low abundance taxa, 88 genera were identified in our samples. To determine the overall microbial composition of each sample, alpha diversity was measured using observed richness and Shannon’s entropy (Figure 3). Generally, XN exposure reduced alpha diversity measures in both SHIME models. XN significantly reduced observed richness in the TC reactor (*p* = 0.01 and *p* = 0.002, T2 and T3, respectively) of the H-SHIME, and the DC reactor (*p* = 0.03 and *p* = 0.04, T2 and T3, respectively) of the D-SHIME. A significant decrease in Shannon diversity was observed in the TC reactor of the H-SHIME (*p* = 0.05). Shannon diversity accounts for both the number of taxa and their relative abundance. This suggests taxa richness and evenness decreased in the healthy microbial community only, while observed richness of both communities decreased with XN treatment. A broader overview of relative abundance changes at the Family level within each SHIME system is provided in Supplementary Figure S1. 

**Figure 4:**
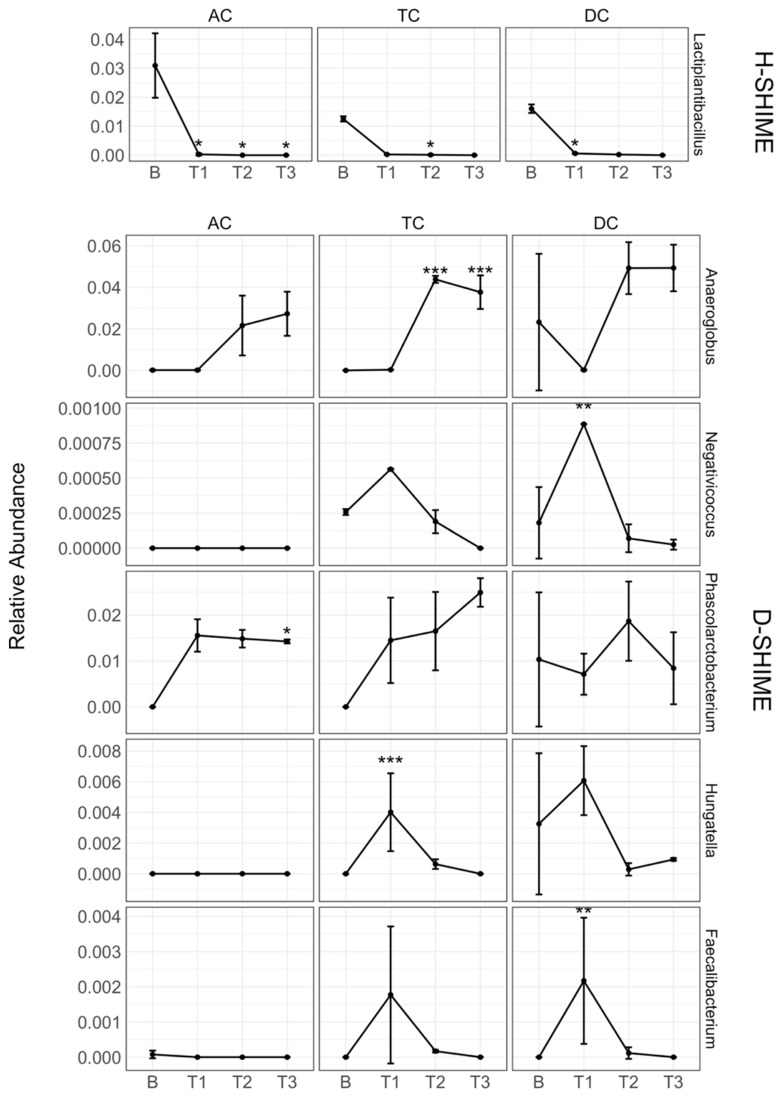
Abundance of bacterial genera changing over time in response to XN exposure and by SHIME model. Taxa were first agglomerated to the genus level. Count data were rarefied and center log-ratio transformed prior to statistical comparisons. The Bonferroni multiple testing correction was used to control for the Family-Wise Error Rate. Measurements were taken in triplicate. Error bars represent the standard deviation. AC: ascending colon; TC: transverse colon; DC: descending colon; B: baseline; T1: treatment week 1; T2: treatment week 2; T3: treatment week 3. *, *p* ≤ 0.05; **, *p* ≤ 0.01; ***, *p* ≤ 0.001.

To explore the effect of XN on individual genera, we conducted a differential abundance analysis. Overall, 6 genera were differentially abundant among both systems following XN exposure (Figure 4). In the H-SHIME, one genus, *Lactiplantibacilllus*, was significantly reduced at T1, T2, and T3 in the AC (*p* = 0.04, *p* = 0.004 and *p* = 0.03, respectively), T2 in the TC (*p* = 0.04), and T1 in the DC (*p* = 0.03) compared to baseline. A similar but nonsignificant trend was noted in the TC. In the D-SHIME, 5 genera were differentially abundant with XN exposure. Interestingly, we observed two shared temporal trends among differentially abundant taxa. In one trend, *Negativicoccus* (DC), *Hungatella* (TC), and *Faecalibacterium* (DC) were significantly more abundant at T1 (*p* = 0.003, *p* < 0.001, and *p* = 0.003, respectively) compared to baseline. Similar temporal trends were observed in TC and DC compartments for all taxa. The relative abundance of these taxa returned to near-baseline levels by T3, suggesting that XN exposure may induce a rapid but transient increase in their growth. In the second trend, *Anaeroglobus* (*p* < 0.001 and *p* < 0.001 at T2 and T3, respectively) in the TC and *Phascolarctobacterium* (*p* = 0.02 at T3) in the AC increased in relative abundance compared to baseline. Similar non-significant temporal trends were also observed in other colon compartments. These time trends in microbiota abundance appear to be inversely related, which may reflect niche competition or cross-feeding dependencies where the expansion of one taxon is associated with the decline of the other.

### 2.4. XN Supplementation Inhibits Bile Acid Metabolism Within the Digestive Meta-Metabolome

**Figure 5:**
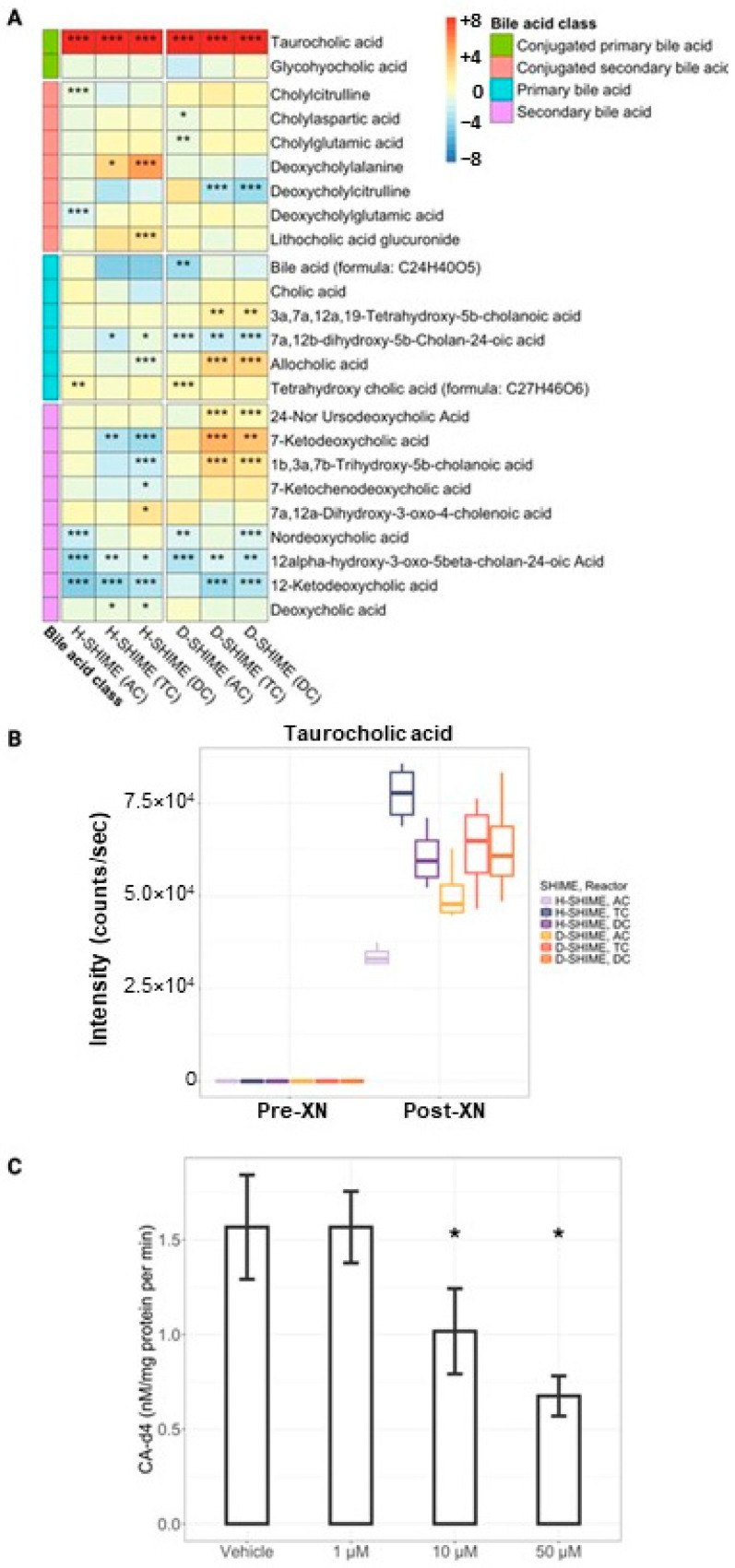
XN exposure disrupts microbiota-derived bile acid metabolism. (**A**) Heatmap of bile acid metabolites. The cell color represents the log 2-fold change in intensity compared to baseline, and the colored block annotations represent the bile acid class of each metabolite. (**B**) Taurocholic acid (TCA) levels by reactor vessel. TCA significantly accumulates across all colon compartments of both SHIME models in response to XN exposure. Each color represents a colon reactor. Purple colors denote H-SHIME reactors. Orange colors denote D-SHIME reactors. (**C**) XN concentration-dependent decrease in enzymatic conversion of TCA into CA via inhibition of fecal bile salt hydrolase (BSH). Fecal BSH enzymes were extracted from stool by sonication and incubated with labeled taurocholic acid-d4 (TCA-d4) and increasing concentrations of XN. Each incubation was performed in triplicate. BSH activity was measured by the rate of cholic acid-d4 (CA-d4) generation. For comparison, the concentration of XN was ~408 µM in SHIME colon reactors during treatment period. AC: ascending colon; TC: transverse colon; DC: descending colon. *, *p* ≤ 0.05; **, *p* ≤ 0.01; ***, *p* ≤ 0.001.

To describe the impact of XN treatment on the digestive metabolome, we conducted an untargeted metabolomics analysis. Among the features detected, 299 were annotated (Supplemental Table S1). Many were found to be di- and tri- oligopeptides. As these may be artifacts of incomplete protein digestion, they were removed from further analysis, as well as metabolites associated with XN. Within the H-SHIME, 42, 37, and 35 annotated metabolites were significantly different with XN treatment in AC, TC, and DC reactors, respectively. Within the D-SHIME, 8, 32, and 74 annotated features were significantly different with XN treatment in AC, TC, and DC reactors, respectively. Metabolites were classified in chemical groups by Chemical Similarity Enrichment Analysis using chemical ontologies and structural similarities. This analysis revealed several classes of bile acids, including cholic acids and glycodeoxycholic acids, as well as amino acids and their degradation products, including acidic amino acids, aromatic amino acids, imidazoles, coumaric acids, ortho-aminobenzoates, phenols, and indoles. While amino acid products were not consistently changed with XN, similarities were observed with bile acid groups between SHIME models. Possibly in relation to disrupted amino acid metabolism, several amino acid-conjugated bile acids were detected, which have been recently identified as bacterial metabolites (categorized as “conjugated secondary bile acid” in Figure 5A) [23]. Most notably, an equal or greater than 10-fold increase in taurocholic acid (TCA) was observed among both SHIME models and all colon reactors (Figure 5B). Additionally, several secondary bile acids, which would result from the deconjugation of TCA and further biotransformation were significantly reduced, suggesting XN interferes with the microbial pathway responsible for TCA metabolism.

### 2.5. XN Inhibits Bile Salt Hydrolase Activity

Based on the findings from the untargeted metabolomics experiment, we generated and tested the hypothesis that the XN-associated increase in TCA levels is caused by XN’s capability to inhibit the activity of bacterial bile salt hydrolase (BSH) enzymes. Bacterial BSH enzymes are responsible for the deconjugation of bile salts; specifically, the deconjugation of taurocholic acid to cholic acid (CA). Total bacterial enzymes were first extracted from fecal material by sonication and incubated with heavy-labeled taurocholic acid (TCA-d_4_) and increasing concentrations of XN. The rate of CA-d_4_ generation was used to monitor the activity of BSH in the presence of XN (Figure 5C). We utilized increasing concentrations of XN to determine the dose-inhibition relationship. We found incubation with increasing concentrations of XN induced a concentration-dependent inhibition in BSH activity. Ten μM (*p* = 0.05) and fifty μM (*p* = 0.02) significantly inhibited CA-d_4_ formation compared to vehicle control (DMSO). Fifty μM XN inhibited over 50% of activity in a mixture of fecal BSH enzymes—a relatively low concentration compared to the ~408 μM XN present in each SHIME reactor—indicating that BSH inhibition may occur at easily achievable physiological concentrations.

## 3. Discussion

In this study, we investigated the effect of XN supplementation on the digestive metabolome of gut microbiota in eubiotic and dysbiotic states utilizing the long-term SHIME system. We identified microbial metabolites of XN and demonstrated that C-C cleavage of XN into simple phenolic acids did not occur in this model. Certain dihydrochalcones can be converted into their corresponding phenylpropionic acids by the C-C cleaving enzyme, phloretin hydrolase, expressed by gut microbiota such as *Eubacterium ramulus* [24]. In previous work conducted by Paraiso et al. [18], no evidence was found that *E. ramulus* converts XN into phenylpropionic acid. The lack of detectable formation of the putative C-C cleavage products in these experiments suggests that XN is a poor substrate for gut bacterial phloretin hydrolase.

We observed XN generally decreased production of SCFAs and alpha diversity. SCFAs are markers of saccharolytic fermentation. Initial SCFA concentrations varied between models, likely reflecting commonly reported differences with dysbiosis in Crohn’s disease [25]. XN reduced acetic acid in both models, primarily in the DC. Additionally, propionic acid and butyric acid formation was dampened in the H-SHIME across all colon compartments. As these SCFAs are produced by fewer taxonomic niches compared to acetic acid, this effect may reflect the greater alpha diversity of the H-SHIME. In fact, alpha diversity, including observed richness and Shannon’s entropy, were significantly reduced in the TC of the H-SHIME. Conversely, only observed richness was significantly reduced in the D-SHIME accompanying acetic acid reductions, suggesting a broader dampening of microbial diversity and function in this system. 

We observed significant changes in the digestive metabolome, especially in the biotransformation of bile acids. Most notably, taurocholic acid (TCA) increased with concurrent decreases in secondary bile acids, reflecting BSH inhibition. Though some BSH enzymes have greater or exclusive substrate specificity for taurine-conjugated bile salts due to a larger and more positively charged binding pocket, future investigations should explore the specificity of XN inhibition towards specific BSH enzymes [26,27]. Inhibition of BSH activity represents a promising therapeutic strategy for restoring bile acid homeostasis in certain chronic diseases. A diminished and dysregulated bile acid pool is a common feature of inflammatory bowel disease (IBD), metabolic syndrome, and non-alcoholic fatty liver disease (NAFLD), often driven by a combination of intestinal inflammation, altered gut microbiota composition, and dietary factors [28,29,30]. Bile acids, beyond their classical role in lipid digestion and absorption, are key regulators for host metabolism, intestinal inflammation, and gut microbiome composition through bile acid receptors (BARs), such as FXR [31,32,33]. Bile acid resorption from the intestine is receptor-mediated and specific to the conjugated form; premature bacterial deconjugation in the upper small intestine—driven by gut microbiome dysbiosis—can disrupt enterohepatic circulation and reduce critical signaling through BARs [34,35]. Additionally, the reduction in secondary bile acids downstream of BSH inhibition may be beneficial as their accumulation in the colon can be cytotoxic at high concentrations, contributing to mucosal injury and inflammation [36,37]. Additionally, excess taurine released from TCA promotes growth of sulfur-reducing bacteria, contributing to excess hydrogen sulfide, which may exacerbate mucosal inflammation in certain conditions like IBD [38]. Bile acid homeostasis is tightly regulated, and disruptions—whether due to excess or deficiency—can exacerbate disease symptoms. Therefore, XN’s bile acid targeted effects warrant attention in future investigation within distinct disease contexts.

Our study presents novel insights into the interactions between XN and the gut microbiota, specifically disrupting bacterial bile acid metabolism through inhibition of BSH. Additionally, this study provides context to dose-specific inhibition of BSH enzymes that can inform future mechanistic and clinical research within the context of specific disease states. The SHIME system provides a valuable platform for isolating the role of the gut microbiota and studying microbiota–xenobiotic interactions within defined colonic compartments [20,21]. Unlike single-tube fecal incubations, SHIME has the important advantage that it can model specific biotic states and microbial metabolism over a period of weeks. However, SHIME experiments have several limitations. It inherently lacks host physiological components that are crucial for fully understanding XN’s mechanisms in vivo. For instance, significant alterations in the bile acid pool may modulate the host immune response, which in turn can shape microbiota composition and function—interactions that are not captured in this model [31,32,33]. Additionally, the system’s low throughput and representation of only a limited number of gut communities constrain its generalizability to the broader human population. While we focus on robust effects consistent between SHIME models, the reliance on single donors limits the taxonomic and metabolic shifts observed to these specific communities. Lastly, the absence of detailed participant information beyond age range and antibiotic usage history constrains our ability to contextualize our findings. Characteristics such as body mass index, race, and ethnicity are increasingly recognized as key determinants of gut microbial composition, activity, and dynamics, and thus may influence responses to dietary polyphenols such as XN. Larger studies with diverse human gut microbiomes will be necessary to establish broader applicability and expand upon these findings. Nevertheless, these SHIME experiments provide us with a plausible hypothesis that we tested in follow-up experiments: XN inhibits gut microbial BSH enzymes, thereby altering microbial bile acid metabolism and homeostasis. The present findings may inform the design of future research that integrates host factors and diverse human gut microbiomes. Larger-scale studies will be essential to understand XN’s therapeutic potential for chronic conditions that involve dysregulated bile acid metabolism.

## 4. Materials and Methods

### 4.1. Fecal Microbiota Sample Collection

Fecal samples for inoculation of the SHIME models were procured from Lee BioSolutions (Maryland Heights, MO, USA), collected in air-tight glass containers with an anaerobic gas generating sachet (BD GasPak, Franklin Lakes, NJ, USA) to limit exposure to oxygen, and prepared within 24 h of collection, following established protocols [20,39]. The H-SHIME model was inoculated with fecal material provided by a healthy woman. The D-SHIME model was inoculated with fecal material provided by a woman diagnosed with Crohn’s Disease. Both women were in the age range 21–50 years old and had no history of antibiotic use 6 months prior to sample collection. Fecal slurries were prepared by making a 20% solution (*w*/*v*) with an anaerobic phosphate buffer (K_2_HPO_4_ 8.8 g/L; KH_2_PO_4_ 6.8 g/L; sodium thioglycolate 0.1 g/L; sodium dithionite 0.015 g/L; flushed with N_2_ for 15 min) and gently homogenizing for 10 min. The fecal slurries were centrifuged (500× *g* for 2 min) to remove large particulates.

### 4.2. Long-Term Colonic Incubation

Two side-by-side SHIME^®^ (ProDigest, Ghent, Belgium) systems consisted of a stomach (ST), small intestine (SI), ascending colon (AC), transverse colon (TC), and descending colon (DC) reactors were configured following an established protocol [20,39]. Briefly, double-jacketed glass vessels were kept at a consistent temperature of 37 °C and contents of each vessel were continuously stirred. Anaerobic conditions were achieved by flushing all reactors with N_2_ daily. Each SHIME colon reactor (AC, TC, and DC) was inoculated at 5% (*v*/*v*) with the respective fecal slurry. Microbial communities were allowed to stabilize for a 2-week period and monitored for short-chain fatty acid production and consumption of HCl (0.5 M) or NaOH (0.5 M) to stabilize pH. The system operated according to a fill-and-draw principle, with peristaltic pumps adding pre-defined volumes of nutritional media to the gastric vessel (ST = 105 mL; pH 2; 25 min transfer time), followed by the mixing of pancreatic juice and bile salts (NaHCO_3_ 2.6 g/L, Oxgall 4.8 g/L and pancreatin 1.9 g/L) in the small intestine vessel (SI = 45 mL; pH 6.5; 12 min transfer time). After 90 min of magnetic stirring, the intestinal suspension was pumped to the ascending colon reactor (AC = 375 mL total volume retained; pH 5.7–5.9; 85 min transfer time), and subsequently to the transverse colon reactor (TC = 600 mL total volume retained; pH 6.2–6.4; 95 min transfer time), descending colon reactor (DC = 450 mL total volume retained; pH 6.6–6.9; 105 min transfer time), and waste container, such that volumes of colon reactors were maintained constant throughout the experiment. The pH of each colon reactor was continuously monitored and automatically adjusted through administration of either HCl (0.5 M) or NaOH (0.5 M) as needed.

### 4.3. Experimental Design and Dosage Information

Following stabilization, a 2-week control period was used as a baseline for microorganism community composition and activity. Following baseline, XN (99+% purity, provided by Hopsteiner, Inc., New York, NY, USA) was administered at 200 mg per day as continuous dosing mixed into nutritional media utilizing our OPT formulation formulation (oleic acid/propylene glycol/Tween 80, 0.9:1:1 *w*/*w*/*w*) as previously reported [11]. The dose of XN was selected based on the human equivalent dose derived from preclinical studies in high-fat-fed mice, as well as human clinical studies utilizing XN as a dietary supplement [4]. Samples were collected 3 times per week at 1130 h, prior to a feed cycle entering the colon reactors to minimize variation introduced by metabolic fluxes.

### 4.4. Quantification of XN Microbial Metabolites

XN and XN microbial metabolites, including isoxanthohumol (IXN), α,β-dihydroxanthohumol (DXN), 6-prenylnaringenin (6PN), 8-prenylnaringenin (8PN), and *O*-desmethyl-α,β-dihydroxanthohumol (DDXN), were quantified by LC-SRM-MS/MS. Two volumes of SHIME fermentation sample were separately prepared for quantification of XN and the XN metabolites. For XN quantification, 10 µL SHIME fermentation sample was first diluted by adding 130 µL 70% ice cold methanol (with 1% ascorbic acid *w*/*w*), 20 µL ^13^C_3_-XN internal standard (100 ng/mL in acetonitrile), and 50 µL MilliQ water. Mixtures were vortexed and metabolites were recovered by liquid–liquid extraction 3 times with 200 µL methyl tert-butyl ether (MTBE). The organic layer was collected, evaporated under reduced pressure in a vacuum desiccator, and reconstituted in 100 µL 50% acetonitrile: water. For quantification of XN metabolites, 100 µL of SHIME fermentation sample was prepared using the same amounts of solvent, internal standard, and water. Similarly, mixtures were extracted 3 times with 200 µL of MTBE, evaporated and reconstituted in 100 µL 50% acetonitrile:water. All samples were analyzed on a Xevo TQ-XS triple quadruple mass spectrometer (Waters, Milford, MA, USA) operating in SRM mode with negative electrospray ionization, coupled with an ACQUITY UPLC I-Class Plus system using an Agilent Zorbax 300SB-C8 column (2.1 × 50 mm, 3.5 µm). The solvent gradient, composed of water (A) and acetonitrile (B), both acidified with 0.1% (*v*/*v*) formic acid, was as follows: from 0 to 0.5 min, 5% B; from 0.5 to 3 min, B increased to 100%; for 1 min, B was held at 100%; and the column was allowed to re-equilibrate at 5% B before the next sample injection. The injection volume was 5 µL and the mobile phase flow was 0.4 mL min^−1^. SRM transitions for quantification were *m*/*z* 353→119, *m*/*z* 353→233 for XN and IX; *m*/*z* 339→119, *m*/*z* 339→219 for 8PN and 6PN; *m*/*z* 355→191, *m*/*z* 355→249 for DXN; and *m*/*z* 341→191, *m*/*z* 341→235 for DDXN.

Utilizing 3-nitrophenyl hydrazine (3NPH) as the derivatization agent and the method described below for short-chain fatty acids, the following standards were derivatized and investigated as potential metabolites of XN: 4-hydroxyphenylpropionic acid (4HPPA), 4-hyroxyphenylacetic acid (4HPAA), and 4-hydroxybenzoic acid (4-HBA). SRM transitions for quantification were *m*/*z* 300→137 (4HPPA-3NPH), *m*/*z* 286→137 (4HPAA-3NPH), and *m*/*z* 272→137 (4HBA-3NPH).

### 4.5. Quantification of Short-Chain Fatty Acids

SCFA were quantified (LC-SRM-MS/MS) as previously described [22] following a method adapted from Han et al. [40], which utilizes 3-nitrophenyl hydrazine (3NPH) to convert SCFAs into their corresponding hydrazides. Authentic standards were used to construct calibration curves for acetate, propionate, and butyrate.

### 4.6. Bacterial 16S rRNA Gene Sequencing, Data Management, and ASV Normalization

Samples (1.5 mL) were collected from each colon reactor and microbial cells were pelleted by centrifugation (5000× *g* for 10 min). The supernatant was removed and stored for later metabolite analyses. The remaining pellet was used to isolate microbial DNA using a QIAamp PowerFecal kit (Qiagen, Germantown, MD, USA) per the manufacturer’s instruction. Bacterial DNA was amplified by PCR using the V4 region of the 16S rRNA gene and then sequenced on an Illumina MiSeq platform to produce a sequence library follow the Earth Microbiome Project protocol [41]. This approach yielded 250 bp paired-end amplicon sequences at a target sequencing depth of 50,000 reads per sample. Library preparation and 16S amplicon sequencing were performed at Pacific Northwest National Laboratories (Richland, WA, USA) using established methods [42]. Data preprocessing, identification of amplicon sequence variants (ASVs), and ASV annotation were completed using the DADA2 pipeline, as implemented in R (v 4.4.1) [43]. Briefly, reads were first trimmed for read quality and filtered for expected errors, merged by paired reads and filtered for chimera ASVs. Finally, taxonomy was assigned using the Silva database (v132) with the Naïve Bayesian classifier built into the DADA2 algorithm [44]. Data were then structured as a Phyloseq object for downstream analysis in R statistical software [45]. Alpha-diversity measures were performed on pre-filtered data using observed richness and Shannon entropy. For differential abundance, taxa were agglomerated to the genus level prior to filtering sparse genera, which included those that were observed fewer than 3 times in at least 20% of the samples and those with a mean relative abundance of less than 0.001% across all samples. This reduced the total observed ASVs from 718 to 88.

### 4.7. Metabolomics

Metabolites were extracted using ice cold methanol (100 μL culture medium/100 μL methanol), vortexed for 2 min, and clarified by centrifugation (13,000 rpm, 10 min). The supernatants were collected and transferred to autosampler vials, prior to analysis on our metabolomics platform, as previously described [46,47,48]. Briefly, HPLC was performed using a Shimadzu Nexera system using a phenyl-3 stationary phase column (Inertsil Phenyl-3, 5 μM, 4.6 × 150 mm, GL Sciences, Torrance, CA, USA) coupled to a quadrupole time-of-flight mass spectrometer (AB Sciex TripleTOF 5600, Concord, Ontario, Canada). Samples were randomized, with auto-calibration performed every two samples, and quality control (QC) samples, composed of a pooled aliquot of each sample, analyzed every 10 samples. Tandem MS spectra in positive and negative ion mode were obtained for all samples using information-dependent acquisition (IDA), while sequential window acquisition of all theoretical spectra (SWATH) was performed on quality control samples. Spectral processing was performed using Progenesis QI (NonLinear Dynamics v2.4, Waters, Durham, NC, USA). Declustering of mass spectra considered the following adducts for positive ion mode, [M + H]^+^, [M + Na]^+^, and [M + NH_4_]^+^, and the following adducts for negative ion mode, [M − H]^−^, [M + FA − H]^−^, and [M − H_2_O − H]^−^. Feature intensities were normalized to all compounds in Progenesis QI. Spectral features with high technical variation, defined as a CV greater than 50% in QC samples, were removed from the data, resulting in 9024 features in positive ion mode and 3543 features in negative ion mode.

A combination of methods was used for feature annotation utilizing PeakView with XIC Manager 1.2.0 (AB Sciex) and Progenesis QI (Waters, Durham, NC, USA). Level 1 and 2 metabolite annotation were assigned based on confidence of annotation [49]. Level 1 annotations were determined by matching accurate mass (error < 6 ppm), retention time (error < 10%), MS/MS fragmentation score (>70), and isotope distribution (error < 20%) with our in-house library of 650 commercially available standards (IROA Technology, Bolton, MA, USA). These annotations were then integrated into the Progenesis QI software (v2.4), where additional Level 2 metabolite annotations were determined by referencing the following online spectral databases: METLIN, Human Metabolome Database (HMDB), ChemSpider, and Microbial Metabolite Database (MMDB). Accepted annotations were based on accurate mass similarity (ppm ≤ 7), isotope similarity (≥70%), and fragmentation score (≥40). Putative metabolite assignments can be found in Supplemental Materials (Supplemental Table S1), along with molecular formula, retention time, monoisotopic ion mass, adducts, mass error, library source for identification, and PubChem ID (PCID). Features were normalized by logarithmic transformation and pareto scaled after zero values were imputed with 1% of the lowest intensity detected across all samples. Features were next filtered by accepted annotations. Di- and tripeptides were removed for clarity as they were likely the result from incomplete protein digestion. Data from positive and negative ion modes were combined and where features were found in both datasets, they were filtered by whichever had the lowest CV within QC samples.

### 4.8. Bile Salt Hydrolase (BSH) Activity

Fecal proteins were extracted from fecal samples (0.5 g) in pH 7.4 PBS (5 mL) using sonication [50]. Fecal protein concentration was quantified using the Pierce Bicinchoninic Acid (BCA) protein assay (Thermo Fisher Scientific, Rockford, IL, USA). BSH activity was measured based on the generation of cholic acid-d_4_ (CA-d_4_) from taurocholic acid-d4 (TCA-d_4_) by fecal proteins. Briefly, the incubation was carried out in 3 mM sodium acetate buffer (pH 5.2) containing 0.1 mg mL^−1^ fecal proteins and 50 µM TCA-d_4_ in a final volume of 200 µL. XN was tested at 1, 10, and 50 µM concentrations. After 20 min at 37 °C, the reaction was terminated by adding equal parts (200 µL) acetonitrile. After centrifugation (15,000 rpm for 10 min at 4 °C), supernatant was transferred to autosampler vials for LC-MS/MS analysis.

### 4.9. LC-SRM-MS/MS Analysis of Cholic Acid-d_4_ Generation

UPLC was performed with an ACQUITY UPLC I-Class Plus system using an ACQUITY BEH C18 column (2.1 × 100 mm, 1.7 µm, Waters, Taunton, MA, USA) coupled to a Xevo TQ-XS triple quadruple mass spectrometer (Waters, Milford, MA, USA) operating in negative electrospray ionization mode. The solvent gradient, composed of water (A) and acetonitrile (B), both acidified with 0.01% (*v*/*v*) formic acid, was as follows: from 0 to 0.5 min, 5% B; from 0.5 to 4 min, B increased to 30%; from 4 to 6 min, B was held at 30%; for 1 min, B increased to 38%; from 7 to 9 min, B increased from 38% to 45%; from 9 to 12 min, B increased to 70%; from 12 to 14, B increased to 100%; for 1 min, the column was returned to 5% B, and equilibrated before the next injection. The injection volume was 5 µL and the mobile phase flow rate was 0.45 mL min^−1^. Taurocholic acid-d4 (TCA-d_4_) and cholic acid-d4 (CA-d_4_) were identified by matching their retention time, molecular [M-H]^−^ ions, and fragmentation pattern to those of the corresponding, non-deuterated isotopologues. The formation of CA-d_4_ was calculated from a calibration curve using CA as the internal standard.

### 4.10. Statistical Analysis

All samples were analyzed in triplicate. All data are expressed as the mean value ± SD. Statistical analyses were performed using R statistical software (v 4.4.1). One-way ANOVA followed by *post hoc* Bonferroni analysis evaluated differences between experimental stages. Model assumptions were tested using Anderson-Darling test for normality and the Levene’s test for homogeneity of variances. When raw data failed these tests, data were transformed using the natural logarithm. Statistical significance was accepted at an adjusted *p* ≤ 0.05. Differential abundance for each taxon before and after XN was assessed following a center-log ratio (clr) transformation after all samples were agglomerated to the genus level and rarefied to an even depth. A comparison was made for each genus. The Bonferroni procedure was used to correct for the Family-Wise Error Rate. While more conservative than False Discovery Rate (FDR) approaches, this method was selected to ensure strict control of false positives and enhance confidence in observed effects with the SHIME models, which are inherently limited in generalizability.

### 4.11. Data and Code Availability

R code for analysis and figure curation is available at GitHub: “jamiesop/SHIME_XN_Microbiome_Analysis”. Raw 16S rRNA gene sequencing reads are available in the NCBI SRA under BioProject PRJNA1183980.

## Data Availability

R code for analysis and figure curation is available at GitHub: “jamiesop/SHIME_XN_Microbiome_Analysis”. Raw 16S rRNA gene sequencing reads are available in the NCBI SRA under BioProject PRJNA1183980.

## Supplementary Materials

The following supporting information can be downloaded at: https://www.mdpi.com/article/10.3390/ijms262110698/s1.

## Abbreviations

XN: xanthohumol; IBD: inflammatory bowel disease; SCFA: short-chain fatty acid; SHIME: Simulator of Human Intestinal Microbial Ecosystem; AC: ascending colon; TC: transverse colon; DC: descending colon; IXN: Isoxanthohumol; DXN: ɑ,β-dihydroxanthohumol; 8PN: 8-prenylnaringenin; DDXN: O-desmethyl-ɑ,β-dihydroxanthohumol; 6PN: 6-prenylnaringenin; ASV: amplicon sequence variant; BSH: bile salt hydrolase; BCA: Bicinchoninic Acid; TCA: taurocholic acid; CA: cholic acid; BAR: bile acid receptor; FXR: farnesoid X receptor; OPT: oleic acid/ propylene glycol/ Tween 80.
