# Supplementary figures and images for: Xanthohumol Alters Gut Microbiota Metabolism and Bile Acid Dynamics in Gastrointestinal Simulation Models of Eubiotic and Dysbiotic States
Source: Int J Mol Sci. 2025 Nov 3;26(21):10698. doi: 10.3390/ijms262110698 (PMC12608232; doi:10.3390/ijms262110698)

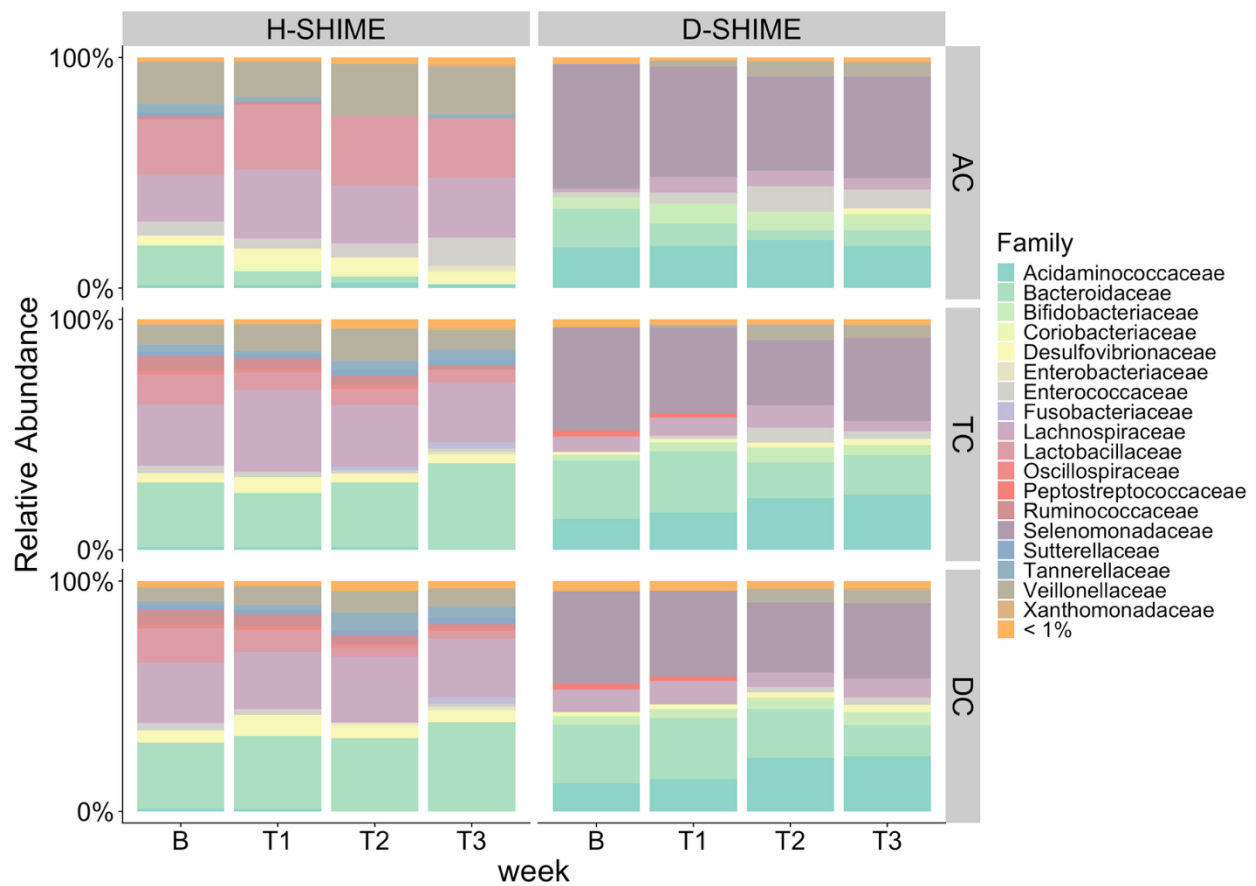

Supplement: Supplementary file 1 [file ijms-26-10698-s001.zip › ijms-3911064-supplementary.pdf]
